# Associations of circadian rest/activity rhythms with cognition in middle-aged and older adults: Demographic and genetic interactions

**DOI:** 10.3389/fnins.2022.952204

**Published:** 2022-10-12

**Authors:** Jill A. Rabinowitz, Yang An, Linchen He, Alfonso J. Alfini, Vadim Zipunnikov, Mark N. Wu, Sarah K. Wanigatunga, Jennifer A. Schrack, Chandra L. Jackson, Luigi Ferrucci, Eleanor M. Simonsick, Susan M. Resnick, Adam P. Spira

**Affiliations:** ^1^Department of Mental Health, Johns Hopkins Bloomberg School of Public Health, Baltimore, MD, United States; ^2^Intramural Research Program, National Institute on Aging, Baltimore, MD, United States; ^3^Department of Environmental Health and Engineering, Johns Hopkins Bloomberg School of Public Health, Baltimore, MD, United States; ^4^National Center on Sleep Disorders Research, Division of Lung Diseases, National Heart, Lung, and Blood Institute, Bethesda, MD, United States; ^5^Department of Neurology and Neurosurgery, Johns Hopkins School of Medicine, Baltimore, MD, United States; ^6^Department of Biostatistics, Johns Hopkins Bloomberg School of Public Health, Baltimore, MD, United States; ^7^Department of Epidemiology, Johns Hopkins Bloomberg School of Public Health, Baltimore, MD, United States; ^8^Center on Aging and Health, Johns Hopkins University, Baltimore, MD, United States; ^9^Epidemiology Branch, Department of Health and Human Services, National Institute of Environmental Health Sciences, National Institutes of Health, Research Triangle Park, Durham, NC, United States; ^10^Intramural Research Program, National Institute on Minority Health and Health Disparities, Bethesda, MD, United States; ^11^Department of Psychiatry and Behavioral Sciences, Johns Hopkins School of Medicine, Baltimore, MD, United States

**Keywords:** circadian rest activity rhythms, actigraphy, cognition, older adults, cognitive decline

## Abstract

**Objectives:**

Wrist actigraphs (accelerometers) can record motor activity over multiple days and nights. The resulting data can be used to quantify 24-h activity profiles, known as circadian rest-activity rhythms (CRARs). Actigraphic CRARs have been tied to cognitive performance and decline in older adults; however, little is known about links between CRARs and performance or change in specific cognitive domains, or how individual differences may influence these associations. We investigated associations of actigraphic CRARs with cognitive performance and change in middle-aged and older adults, and explored whether age, sex/gender, race, and apolipoprotein E (APOE) e4 carrier status moderated these associations.

**Materials and methods:**

Participants (*N* = 422; 47% male) were cognitively healthy adults (i.e., without mild cognitive impairment or dementia) at baseline aged ≥ 50 years from the Baltimore Longitudinal Study of Aging who completed 5.6 ± 0.89 nights of wrist actigraphy and tests of memory, executive function, attention, language, and visuospatial ability at the same visit the actigraph was issued; 292 participants had repeat cognitive testing 3.12 (1.58) years later. Predictors included indices of rhythm strength [i.e., amplitude; relative amplitude (RA); interdaily stability (IS); mesor], delayed timing of the rhythm peak [i.e., later acrophase; midpoint of an individual’s least active 5 h (L5 time); midpoint of an individual’s most active 10 h (M10 time)], and fragmentation [i.e., intradaily variability (IV)].

**Results:**

In main effects, later L5 time was cross sectionally associated with poorer memory, and greater IS predicted slower longitudinal memory decline. Associations of CRARs with cognition differed as a function of age, sex/gender, race, and APOE e4 carrier status.

**Conclusion:**

Among middle-aged and older adults, delayed circadian phase is associated with poorer memory performance, and greater day-to-day rhythm stability is associated with slower declines in memory. Significant interactions suggest that CRARs are generally more strongly associated with cognitive performance and rate of cognitive decline among women, Black adults, older individuals, and APOE e4 carriers. Replication in independent samples is needed.

## Introduction

Numerous studies link sleep characteristics to cognitive performance and subsequent decline in older adults, as well as to diagnoses of mild cognitive impairment (MCI) and dementia (Devore et al., 2016; Shi et al., 2018). Less is known, however, about associations of circadian rhythms with subsequent cognitive decline among cognitively healthy older adults. Many physiological processes (e.g., core body temperature, melatonin, and cortisol secretion) follow a circadian rhythm; they cycle approximately once every 24 h under endogenous control of the suprachiasmatic nucleus (SCN) of the hypothalamus, and are synchronized with the light/dark cycle through environmental cues, including bright light and feeding (Zee et al., 2013; Lananna and Musiek, 2020). Characteristic changes in circadian rhythms occur with aging. Circadian rest/activity rhythms (CRARs), estimated from continuously collected wrist actigraphy data, are routinely used as a proxy measure of circadian rhythms in human research. Aging is associated with decreases in CRAR amplitude (i.e., reductions in the magnitude of difference in activity between active and rest phases) and phase advances (i.e., earlier daily peaks in activity), both of which may have consequences for cognitive functioning in later life (Krishnan and Lyons, 2015; Musiek et al., 2018) [Definitions of standard CRAR metrics are presented in Table 1].

**TABLE 1:** Definitions of circadian rest activity rhythm (CRAR) indices.

	Definition
**Cosinor indices**	
Amplitude	Difference between the minimum and maximum activity value
Mesor	Average activity over 24 h
Acrophase	Time of maximum activity counts over 24 h
**Non-parametric indices**	
Relative amplitude (RA)	Metric of rhythm strength; normalized difference between most active 10 h and least active 5 h
Interdaily stability (IS)	Stability of activity profiles over days
Intradaily variability (IV)	Fragmentation of the rhythm relative to its 24-h amplitude
L5 time	Midpoint of an individual’s L5 interval
M10 time	Midpoint of an individual’s M10 interval

Although several cross-sectional studies have linked altered CRARs to cognitive function in older adults (Krishnan and Lyons, 2015), findings have been mixed. For example, Oosterman et al. (2009), Luik et al. (2015), and Yang et al. (2022) found that greater rhythm fragmentation [intradaily variability (IV)] was associated with poorer global cognition and performance on tests of executive function, although Yang et al. (2022) also observed a negative association between IV and memory performance. Oosterman et al. (2009) and Yang et al. (2022) also found that weaker CRARs (i.e., lower amplitude and relative amplitude) were associated with poorer performance on tests of memory, executive function, auditory attention, and global cognition. Yi Lee et al. (2021), however, found no association of rhythm strength metrics (i.e., mesor, amplitude) or circadian timing (i.e., acrophase) with dementia or MCI status at baseline. Longitudinal studies examining CRARs with subsequent change in cognitive performance have found that metrics indicative of lower rhythm strength (i.e., mesor, amplitude) and delayed rhythm timing (i.e., later acrophase) were associated with greater declines in global cognition and executive function (Walsh et al., 2014; Rogers-Soeder et al., 2018; Yi Lee et al., 2021; Xiao et al., 2022a,b), and increased risk for MCI and dementia (Xiao et al., 2022b). Other work has shown that greater IV at baseline and greater increases in IV were associated with greater declines in global cognition, and that lower RA and IS, and higher IV at baseline was associated with greater risk for developing incident cognitive impairments among older adult men (Xiao et al., 2022a). Li et al. (2020) also found that greater increases in IS and amplitude and slower decreases in IV were associated with slower global cognitive decline. Taken together, the majority of the literature has linked greater CRAR fragmentation, weaker rhythm strength, and greater phase delay with greater risk for poor cognitive performance and faster cognitive decline.

In the present study, we sought to address several gaps in the literature. First, most longitudinal studies of CRAR-cognition associations have examined a single measure of global cognitive function and, at most, one other cognitive measure. The examination of associations of CRARs with multiple cognitive domains has the potential to elucidate associations that might be obscured when using a global cognitive composite. Second, few studies have examined associations of cognitive outcomes with both parametric cosinor and non-parametric CRAR metrics. Whereas parametric cosinor approaches assume that CRARs can be adequately modeled with a cosine function, non-parametric methods do not make such distributional assumptions and can be a more flexible modeling approach. Third, no studies to our knowledge have evaluated whether age, sex, race, and apolipoprotein E (APOE) genotype modify associations between CRARs and cognitive function or change. Women (Mazure and Swendsen, 2016), people of color (Mayeda et al., 2016), older age groups (Xia et al., 2018), and APOE e4 allele carriers (Wolfe et al., 2018) are at heightened risk for MCI and dementia. However, the extent to which these demographic and genetic factors interact with CRAR characteristics to influence their association with cognitive health remains unknown.

To address the aforementioned gaps, we investigated associations of CRARs (including standard parametric cosinor and non-parametric metrics) with cognitive performance and change in cognitive performance over time in five cognitive domains in middle-aged and older adults who were cognitively normal at baseline. We hypothesized that weaker, more fragmented, less stable, and more phase-delayed rhythms would be associated with poorer cognitive performance at baseline and greater decline in performance over time. We also explored whether these associations differed as a function of participant age, sex, race, and APOE e4 allele carrier status.

## Materials and methods

### Participants

We studied participants in the Baltimore Longitudinal Study of Aging (BLSA), an observational study aimed at understanding cognitive and physical health changes that occur with aging (Shock et al., 1984). BLSA is an ongoing study that continuously recruits healthy adults ≥ 20 years of age. Adults aged < 60 years are scheduled for a follow-up visit every 4 years, those aged 60–80 years are scheduled for follow-up visits every 2 years, and those > 80 years of age are scheduled for annual follow-up visits. At enrollment and subsequent visits, participants provided written informed consent. All study protocols were approved by the Institutional Review Board of the Intramural Research Program of the National Institutes of Health.

We restricted our sample to 435 adults ≥ 50 years of age who completed wrist actigraphy and cognitive assessments at one or more study visits between 2012 and 2019 (Shock et al., 1984). We defined our study “baseline” as the first study visit at which participants had both actigraphy and cognitive data. We excluded a total of 13 participants with either dementia, MCI, or cognitive impairment not meeting MCI criteria at baseline, as determined by previously described cognitive adjudication procedures (Williams et al., 2019), yielding an analytic sample of *N* = 422.

### Wrist actigraphy

Participants were asked to wear an actigraph (Actiwatch-2, Philips-Respironics, Bend, OR, United States) on their non-dominant wrist for seven 24-h periods. This device logs movement in activity counts and records ambient light levels using a built-in accelerometer and photometer, respectively. While wearing the actigraph, participants were instructed to complete sleep diaries each morning and evening, in which they recorded information including the times they got into bed at night with the intention of sleeping (i.e., “lights out”) and when they got up to start their day, and the timing of any naps and any actigraph removal. They were also instructed to press an event-marker button on the watch at lights out and upon getting up to start the day. We used event-marker, sleep diary, and ambient light level data to identify the in-bed intervals, to which we applied a validated algorithm (Kushida et al., 2001) to derive sleep parameters using Actiware v. 6.0.9 software (Philips Respironics).

Actigraphic activity count data were preprocessed using R software Version 4.0.4 (R Core Team, 2020). First, we removed 24-h periods with > 5% (>72 min) of data missing. Second, we log-transformed the activity counts (AC) to create a more symmetric distribution. Third, we averaged the log-transformed AC within 144 distinct 10-min bins. Fourth, we estimated the mean subject-specific rest activity rhythms by averaging the daily curves over the days for which valid actigraphy data were collected. In all analyses, the mean subject-specific rest activity rhythm was reflected in 144, 10-min log-transformed ACs.

### Cosinor circadian rest-activity rhythm metrics

We computed standard parametric cosinor CRAR metrics, which are based on the assumption that CRARs reflect a cosine curve (Ancoli-Israel et al., 2003), using the “cosinor” package^1^ in R (R Core Team, 2020). These included two measures of rhythm strength, namely amplitude (peak-to-trough difference) and mean of estimated cosinor curve (mesor; average activity level), and acrophase (timing of peak activity), a measure of circadian timing (i.e., “phase”) (de Feijter et al., 2020).

### Non-parametric circadian rest-activity rhythm metrics

We also calculated non-parametric circadian indices using the “ActCR” package^2^ in R (R Core Team, 2020), given that CRARs do not always manifest a cosinor (parametric) shape. Specifically, we computed: relative amplitude (RA; rhythm strength); interdaily stability (IS; consistency of rhythm across days); intradaily variability (IV; within-day rhythm fragmentation); midpoint of an individual’s total activity during the least active continuous 5 h (L5 time); and midpoint of an individual’s total activity during the most active continuous 10 h (M10 time) (de Feijter et al., 2020).

### Cognitive measures and domains

Memory, attention, executive function, language, and visuospatial performance were assessed via comprehensive, neuropsychological batteries administered to participants by trained study staff at baseline and subsequent visits (Table 2). For each domain, we created composite scores reflecting the averaged z-scores of each individual test, derived using means and standard deviations of the baseline performances of participants in our sample. For completion times involving the Trail Making Tests Parts A and B, we log-transformed, z-scored, and inverted the signs to ensure that higher scores reflected better cognitive performance before calculating the composite scores.

**TABLE 2:** Cognitive tests in each domain.

Cognitive domain	Cognitive tests
Attention	● Wechsler Adult Intelligence Scale-Revised (WAIS-R) Digit Span Forward Test (Wechsler, 1992) ● Trail Making Test: Part A (Reitan, 1958)
Executive function	● Wechsler Adult Intelligence Scale-Revised (WAIS-R) Digit Span Backward Test (Wechsler, 1992) ● Trail Making Test: Part B (Reitan, 1958)
Memory	● California Verbal Learning Test (CVLT) Immediate Free Recall and Long Delay Recall tests (Delis et al., 1988)
Language	● Letter Fluency (Benton, 1968) ● Categorical Fluency (Newcombe, 1969)
Visuospatial performance	● Clock Drawing Test (Rouleau et al., 1992) ● Modified Version of Educational Testing Service Card Rotations Test (Wilson et al., 1975)

### Other measures

Demographic characteristics including age, sex/gender (i.e., male, female), race, and years of education were obtained via participant self-report. Participant responses on race were categorized as White, Black/African American, and “Other,”—defined here as individuals who identified as either Chinese, Filipino, Japanese, American Indian or Alaskan Native, Other Asian, or Other non-White. Height and weight measurements were assessed at each visit and used to compute body mass index (BMI; kg/m^2^). Participants sleeping pill or medication use was assessed using a single item, “In the past month, how often did you take sleeping pills or other medications to help you sleep?” Response options included “never,” “ <1/week,” “1–2/week,” “3–4/week,” and “5+ times a week.” We dichotomized responses (0 = never, 1 = all other responses). Participants were asked whether they had a history of hypertension, diabetes, congestive heart failure, stroke, peripheral artery disease, angina, or heart attack. Based on these responses, we created a dichotomous cardiovascular disease variable (0 = none, 1 = ≥ 1 condition). We also created a dichotomous smoking status variable; participants who reported that they “never smoked” or “quit smoking at least 10 years prior” were coded as non-smokers and those who reported that they were a “current smoker” or “quit within the past 10 years” were coded as smokers. Depressive symptoms were assessed using the 20-item Center for Epidemiological Studies Depression Scale (CES-D) (Radloff, 1977; Hertzog et al., 1990). To avoid collinearity with our CRAR predictors, we excluded the CES-D item “My sleep was restless” when calculating total scores. Finally, APOE e4 carrier status was determined using two different approaches employed over the course of the BLSA study and dichotomized between yes vs. no. The first approach was employed earlier in the BLSA study and involved using polymerase chain reaction (PCR) amplification with *Hha*I restriction isotyping (Hixson and Vernier, 1990). The second method employed the TaqMan approach, a PCR-based method, that involved leveraging oligonucleotide probes unique to each allele (Koch et al., 2002).

## Statistical analyses

We calculated descriptive statistics for baseline participant characteristics and conducted analyses (e.g., *t*-tests, chi-squared tests) to determine whether there were significant differences in the characteristics of participants with baseline data only, compared to those with longitudinal data. All continuous variables, except time, were z-scored (M = 0, SD = 1) prior to model fitting. To investigate the association of baseline CRARs with cognitive performance at baseline and rate of subsequent change in performance, we fit separate linear mixed-effects models with one cognitive domain composite as the outcome and one cosinor or non-parametric circadian rhythm parameter as the primary predictor.

We fit partially and fully adjusted regression models to examine associations of baseline CRARs with cognitive outcomes. In addition to a CRAR parameter, time of follow up in years, and the interaction between the CRAR parameter and time, Model 1 included baseline age, sex, race, years of education, and interactions between time and each of these covariates. In these models, the main effect of a CRAR parameter reflects the cross-sectional association between the CRAR metric and cognitive performance at baseline; the CRAR parameter*time interaction reflects the longitudinal association of each CRAR metric with change in cognitive performance over time. Model 2 included all variables in Model 1, as well as baseline BMI, cardiovascular disease burden, smoking status, sleep medication use, CES-D score (minus the sleep item), APOE e4 carrier status, and interactions between these covariates and time.

We also examined whether age, sex, race, and APOE e4 carrier status moderated cross-sectional associations of CRARs with cognitive functioning and longitudinal associations of CRAR metrics with cognitive change by entering two-way interactions between each potential moderator and primary predictor variables, and three-way interactions (moderator*primary predictor*time) simultaneously in fully adjusted models. When there was a three-way interaction, all lower-order terms were included in the model. Baseline age was modeled as continuous variable. To understand how age modifies the association, for significant age interactions, we obtained model-derived point estimates of the associations between the primary predictor variable and cognition at ages 65, 75, and 85. Significant interactions were defined as any interaction that yielded a *p*-value of < 0.05, and only results of significant interactions are presented. Because few (*n* = 27) participants reported “Other” race/ethnicity, we removed them from analyses investigating race as a moderator. All linear mixed effects models included intercept and time as random effects with unstructured covariance.

Circadian rest-activity rhythms studies have shown that temporal resolution at which actigraphy data are analyzed can lead to significantly different results (Goncalves et al., 2014). Thus, we conducted sensitivity analyses with mean subject-specific CRARs calculated based on 24 60-min bins, as opposed to the 144 10-min bins used in our main analyses. All analyses were performed in R Version 4.0.4 using the lme() function from “lme4” package (Bates et al., 2015).

## Results

Demographic information for the analytic sample can be found in Table 3. One hundred and 37 participants (30.8%) with actigraphy data had cognitive data at baseline only. Among participants with ≥2 (i.e., repeated) cognitive assessments, the mean number of visits was 3.15 (1.29) (range 2–7) and the mean follow-up time was 3.12 years (1.58) (range 1–7). Participants completed an average of 5.6 nights of actigraphy data (SD = 0.89 days, Median = 6 days; range 3–7 days). Descriptive statistics of CRAR metrics, including cosinor and non-parametric indices, for the whole sample and stratified by sex, race, and APOE e4 status can be found in Table 4. As shown in Table 5, individuals with baseline data only were younger, had lower rates of cardiovascular disease risk factors/morbidity, and performed better on executive function and visuospatial skills tests. Results from the primary analyses are presented below.

**TABLE 3:** Baseline participant characteristics for the whole sample and stratified by sex, race, and APOE e4 status.

	All (*N* = 422)	Sex/Gender		Race			APOE e4^a^ carrier status	
		Male (*n* = 199)	Female (*n* = 223)	White adults (*n* = 302)	Black adults (*n* = 93)	Other race^b^ (*n* = 27)	APOE e4−(*n* = 291)	APOE e4+ (*n* = 103)
Baseline age, in years, M (SD)	72.7 (10.1)	74.1 (10.3)	71.5 (9.8)	74.1 (9.9)	69.3 (9.6)	69.2 (10.9)	73.7 (9.9)	71.4 (9.9)
Sex, N (%)								
Male	199 (47.2%)	–	–	156 (51.7%)	32 (34.4%)	11 (40.7%)	134 (34.3%)	49 (47.6%)
Female	223 (52.8%)	–	–	146 (48.3%)	61 (65.6%)	16 (59.3%)	157 (54.0%)	54 (52.4%)
Race, N (%)								
White adults	303 (71.6%)	156 (78.4%)	146 (65.5%)	–	–	–	221 (75.9%)	58 (56.3%)
Black adults	93 (22.0%)	32 (16.1%)	61 (27.4%)	–	–	–	53 (18.2%)	38 (36.9%)
Other race	27 (6.4%)	11 (5.5%)	16 (7.2%)	–	–	–	17 (5.8%)	7 (6.8%)
Education (in years), M (SD)	17.8 (2.4)	18.0 (2.4)	17.5 (2.4)	17.8 (2.4)	17.3 (2.3)	18.9 (2.8)	17.6 (2.5)	18.1 (2.2)
BMI, M (SD)	27.0 (4.3)	27.4 (3.9)	26.7 (4.7)	26.6 (4.2)	28.7 (4.6)	25.6 (3.4)	27.1 (4.4)	27.1 (4.2)
CES-D Score^c^, M (SD)	3.8 (4.4)	3.8 (4.4)	3.8 (4.4)	3.7 (4.3)	4.5 (4.9)	2.8 (3.2)	4.1 (4.7)	3.3 (3.7)
Sleep Medication Use, N (%)								
No	355 (85.1%)	171 (86.8%)	184 (83.6%)	247 (82.9%)	85 (92.4%)	23 (85.2%)	248 (85.2%)	86 (83.5%)
Yes	62 (14.9%)	26 (13.2%)	36 (16.4%)	51 (17.1%)	7 (7.6%)	4 (14.8%)	43 (14.8%)	17 (16.5%)
Cardiovascular disease risk factors/morbidity, N (%)								
No	194 (46.0%)	82 (41.2%)	112 (50.2%)	161 (53.3%)	22 (23.7%)	11 (40.7%)	133 (45.7%)	41 (39.8%)
Yes	228 (54.0%)	117 (58.8%)	111 (49.8%)	141 (46.7%)	71 (76.3%)	16 (59.3%)	158 (54.3%)	62 (60.2%)
Smoking status, N (%)								
Never smoked or quit > 10 years ago	407 (97.8%)	193 (98.5%)	214 (96.4%)	291 (97.7%)	89 (95.7%)	27 (100%)	280 (96.9%)	101 (99.0%)
Current smoker or quit < 10 years ago	11 (2.6%)	3 (1.5%)	8 (3.6%)	7 (2.3%)	4 (4.3%)	0 (0%)	9 (3.1%)	1 (1.0%)
APOE e4 carrier status, N (%)								
APOE e4−	291 (73.9%)	134 (73.2%)	157 (74.4%)	221 (79.2%)	53 (58.2%)	17 (7**0**.8%)	–	–
APOE e4+	103 (26.1%)	49 (26.8%)	54 (25.6%)	58 (20.7%)	38 (41.8%)	7 (29.2%)	–	–

^a^APOE e4 = apolipoprotein E carrier status.

^b^Other race includes individuals who identified as either Chinese, Filipino, Japanese, American Indian or Alaskan Native, Other Asian, or Other non-White.

^c^CES-D, Center for Epidemiologic Studies Depression Scale score with the sleep item removed.

**TABLE 4:** Baseline CRAR characteristics for the whole sample and stratified by sex, race, and APOE e4 status.

	All (*N* = 422)	Sex		Race			APOE e4 carrier status	
		Male (*n* = 199)	Female (*n* = 223)	White adults (*n* = 302)	Black adults (*n* = 93)	Other adults (*n* = 27)	APOE e4–(*n* = 291)	APOE e4+ (*n* = 103)
**Cosinor metrics, M (SD)**								
Amplitude	2.15 (0.39)	2.04 (0.41)	2.25 (0.34)	2.19 (0.39)	2.08 (0.38)	2.00 (0.41)	2.16 (0.41)	2.13 (0.33)
Mesor	3.23 (0.40)	3.22 (0.40)	3.24 (0.39)	3.23 (0.39)	3.24 (0.41)	3.22 (0.44)	3.22 (0.39)	3.26 (0.42)
Acrophase	2:41:24 (1:07:12)	2:38:24 (1:06:00)	2:44:24 (1:08:24)	2:36:36 (1:02:24)	2:53:24 (1:19:48)	3:00:36 (1:06:36)	2:40:48 (1:06:36)	2:48:00 (1:10:48)
**Non-parametric metrics, M (SD)**								
RA	0.78 (0.09)	0.75 (0.09)	0.80 (0.07)	0.78 (0.09)	0.77 (0.08)	0.76 (0.08)	0.77 (0.09)	0.78 (0.07)
IS	0.66 (0.11)	0.63 (0.12)	0.70 (0.10)	0.67 (0.11)	0.62 (0.11)	0.61 (0.13)	0.66 (0.11)	0.66 (0.11)
IV	0.32 (0.10)	0.35 (0.12)	0.30 (0.09)	0.32 (0.11)	0.33 (0.09)	0.36 (0.12)	0.33 (0.11)	0.32 (0.09)
L5 time	1.53 (1.34)	1.59 (1.56)	1.48 (1.12)	1.48 (1.35)	1.74 (1.37)	1.43 (1.11)	1.54 (1.43)	1.44 (1.04)
M10 time	9.08 (1.42)	9.02 (1.44)	9.15 (1.40)	9.01 (1.38)	9.15 (1.53)	9.70 (1.36)	9.09 (1.45)	9.12 (1.43)

**TABLE 5:** Descriptive statistics for participants with baseline data only vs. baseline and longitudinal data.

Participant characteristics	Participants with baseline data only (*N* = 130)	Participants with baseline + longitudinal data (*N* = 292)	Chi-squared	*t*-value	*P*-value
Baseline Age, in years, M ± SD	69.2 ± 11.7	75.9 ± 8.83	–	−4.45	0.00001
Sex, N (%)			0.98	–	0.321
Male	66 (49.2%)	133 (33.9%)	–	–	–
Female	64 (50.8%)	159 (54.5%)	–	–	–
Race			1.09	–	0.580
White	96 (73.8%)	212 (70.5%)	–	–	–
Black	28 (21.5%)	69 (22.2%)	–	–	–
Other	6 (4.6%)	23 (7.2%)	–	–	–
Baseline educational attainment (in years), M ± SD	17.5 ± 2.20	17.8 ± 2.52	–	−1.58	0.115
Baseline BMI, M ± SD	27.5 ± 4.62	26.6 ± 4.04	–	1.47	0.144
Baseline CES-D Score,^a^ M ± SD	3.91 ± 4.50	3.72 ± 4.26	–	0.35	0.730
Baseline use of sleep medication,^b^ N (%)			0.18	–	0.671
No	105 (84.0%)	250 (85.6%)	–	–	–
Yes	20 (16.0%)	42 (14.4%)	–	–	–
Baseline cardiovascular disease risk factors/morbidity, N (%)			6.70	–	0.010
No	72 (55.0%)	122 (41.8%)	–	–	–
Yes	59 (45.0%)	170 (58.2%)	–	–	–
Baseline smoking status,^b,c^ N (%)			–	–	0.392
Never smoked or quit > 10 years ago	122 (95.3%)	285 (98.3%)	–	–	–
Current smoker or quit < 10 years ago	6 (4.7%)	5 (1.7%)	–	–	–
APOE Carrier Status,^b^ n (%)			0.34	–	0.562
APOE e4−	85 (75.5%)	206 (73.0%)	–	–	–
APOE e4+	27 (22.5%)	76 (27.0%)	–	–	–
Baseline CRARs, M ± SD					
Amplitude	2.18 ± 0.38	2.13 ± 0.40	–	0.81	0.420
Mesor	3.22 ± 0.39	3.22 ± 0.40	–	−0.46	0.648
Acrophase	14.8 ± 1.04	14.7 ± 1.17	–	1.21	0.228
Relative amplitude	0.78 ± 0.08	0.77 ± 0.65	–	1.47	0.143
Intradaily stability	0.11 ± 0.32	0.11 ± 0.33	–	0.19	0.849
Interdaily variability	0.32 ± 0.10	0.33 ± 0.11	–	−0.41	0.684
L5 time	1.54 ± 1.20	1.51 ± 1.37	–	0.11	0.917
M10 time	9.26 ± 1.28	8.97 ± 1.45	–	1.81	0.071
Cognitive outcomes, M ± SD					
Verbal memory	0.11 ± 1.01	−0.08 ± 0.97	–	1.61	0.109
Executive function	0.12 ± 0.89	−0.19 ± 0.72	–	2.17	0.031
Attention	0.13 ± 0.96	−0.16 ± 0.77	–	1.95	0.053
Language	0.11 ± 0.98	−0.11 ± 0.77	–	1.56	0.120
Visuospatial skills	0.22 ± 0.86	−0.13 ± 0.77	–	3.19	0.002

^a^CES-D, Center for Epidemiologic Studies Depression Scale score without the sleep item.

^b^Percentages based on participants with complete data.

^c^Fisher’s test was used to determine differences in smoking status between those with baseline data only vs. longitudinal data. M, mean; SD, standard deviation.

### Memory

In fully adjusted models, later L5 time (i.e., greater phase delay) was cross sectionally associated with poorer memory performance [β = −0.09 (95% CI: 0.16, −0.02), *p*-value = 0.012] (Table 6) and poorer day-to-day stability (i.e., interdaily stability; IS) of rhythms was longitudinally associated with faster memory decline [β = 0.03 (95% CI 0.01, 0.05), *p*-value = 0.004] (Table 6).

**TABLE 6:** Associations of circadian metrics with memory, estimated beta (95% CI).

	Model 1		Model 2	
	Baseline cross-sectional effect	Effect on cognitive change	Baseline cross-sectional effect	Effect on cognitive change
**Cosinor metrics**				
Amplitude	0.02 (−0.06, 0.10)	0.02^†^ (−0.001, 0.03)	0.02 (−0.07, 0.10)	0.02 (−0.003, 0.03)
Mesor	−0.002 (−0.08, 0.07)	−0.0001 (−0.02, 0.02)	−0.001 (−0.08, 0.08)	−0.002 (−0.02, 0.02)
Acrophase	0.03 (−0.05, 0.10)	−0.01 (−0.03, 0.01)	0.02 (−0.06, 0.10)	−0.01 (−0.03, 0.01)
**Non-parametric**				
RA	0.05 (−0.03, 0.12)	0.01 (−0.01, 0.03)	0.05 (−0.03, 0.13)	0.004 (−0.02, 0.02)
IS	0.01 (−0.07, 0.08)	0.03** (0.01, 0.05)	−0.002 (−0.08, 0.08)	0.03** (0.01, 0.05)
IV	0.02 (−0.05, 0.10)	−0.01 (−0.03, 0.01)	0.02 (−0.06, 0.10)	−0.01 (−0.03, 0.01)
L5 time	−0.08* (−0.15, −0.01)	−0.004 (−0.02, 0.01)	−0.09* (−0.16, −0.02)	−0.001 (−0.01, 0.01)
M10 time	–	–	–	–

^†^*p* < 0.10; *p < 0.05; **p < 0.01; – = significant interaction, main effect not reported (see tables below). Model 1 adjusted for baseline age, sex, race, years of education, time, baseline age*time, sex*time, race*time, and years of education*time. Model 2 adjusted for Model 1 covariates, in addition to baseline BMI, sleep medication use, APOE e4 status, depression symptoms (minus the sleep item), cardiovascular disease risk, smoking status, BMI*time, cardiovascular disease risk*time, smoking status*time, sleep medication*time, APOE e4 status*time, and depression symptoms (minus the sleep item)*time.

Longitudinally, there was an interaction of race and M10 with verbal memory (*p*-value for interaction = 0.040) (Table 13). Regression coefficients suggest that a greater M10 time is associated with slower declines in verbal memory among White adults, but greater declines among Black adults, although these point estimates were not significant.

### Language

There were no significant main effects of CRARs with respect to language (Table 7), but we observed many interactions (all *p*-values < 0.05). Model-derived point estimates indicated that higher mesor and lower rhythm fragmentation (i.e., intradaily variability; IV) at age 65 were associated with greater declines in language, but these same CRAR parameters were associated with slower declines in language at age 85 (Table 11). In addition, higher amplitude and IS were associated with slower declines in language in this older age group.

**TABLE 7:** Associations of circadian metrics with language, estimated beta (95% CI).

	Model 1		Model 2	
	Baseline cross-sectional effect	Effect on cognitive change	Baseline cross-sectional effect	Effect on cognitive change
**Cosinor metrics**				
Amplitude	–	–	–	–
Mesor	–	–	–	–
Acrophase	0.01 (−0.05, 0.07)	0.003 (−0.01, 0.01)	0.01 (−0.05, 0.08)	0.003 (−0.01, 0.01)
**Non-parametric**				
RA	0.04 (−0.02, 0.11)	0.002 (−0.01, 0.01)	0.03 (−0.04, 0.09)	0.01 (−0.01, 0.02)
IS	–	–	–	–
IV	–	–	–	–
L5 time	−0.04 (−0.09, 0.01)	0.003 (−0.01, 0.01)	−0.04 (−0.09, 0.01)	0.003 (−0.01, 0.01)
M10 time	–	–	–	–

– = significant interaction, main effect not reported (see tables below). Model 1 adjusted for baseline age, sex, race, years of education, time, baseline age*time, sex*time, race*time, and years of education*time. Model 2 adjusted for Model 1 covariates, in addition to baseline BMI, sleep medication use, APOE e4 status, depression symptoms (minus the sleep item), cardiovascular disease risk, smoking status, BMI*time, cardiovascular disease risk*time, smoking status*time, sleep medication*time, APOE e4 status*time, and depression symptoms (minus the sleep item)*time.

Race moderated the associations of (a) mesor, (b) M10 time, and (c) IV with language longitudinally (all *p*-values for interactions < 0.05) (Table 13). Higher mesor, earlier M10 time, and lower IV were associated with greater declines in language in Black participants but not White participants (Table 13).

Significant interactions were also observed between APOE e4 carrier status and mesor with language cross sectionally (*p*-value for interaction = 0.008) and longitudinally (*p*-value for interaction = 0.024) (Table 14). Higher mesor was associated with better language ability, but greater language declines, in e4 carriers, but not among non-carriers (Table 14).

### Executive function

There were no significant main effects of CRARs with respect to executive function (Table 8). We observed a significant cross-sectional interaction of IS with APOE e4 carrier status (*p*-value for interaction = 0.015) such that higher IS was associated with better executive performance in e4 carriers, but not non-carriers (Table 14).

**TABLE 8:** Associations of circadian metrics with executive function, estimated beta (95% CI).

	Model 1		Model 2	
	Baseline cross-sectional effect	Effect on cognitive change	Baseline cross-sectional effect	Effect on cognitive change
**Cosinor metrics**				
Amplitude	−0.02 (−0.09, 0.04)	0.01†(−0.001, 0.03)	−0.02 (−0.09, 0.05)	0.01 (−0.01, 0.03)
Mesor	−0.05 (−0.11, 0.01)	0.003 (−0.01, 0.02)	−0.05 (−0.12, 0.01)	0.002 (−0.01, 0.02)
Acrophase	0.001 (−0.06, 0.07)	−0.01 (−0.03, 0.003)	0.01 (−0.06, 0.07)	−0.01 (−0.02, 0.01)
**Non-parametric**				
RA	0.03 (−0.04, 0.10)	0.01 (−0.01, 0.02)	0.03 (−0.03, 0.10)	0.003 (−0.01, 0.02)
IS	0.01 (−0.05, 0.08)	0.01 (−0.01, 0.02)	0.02 (−0.04, 0.09)	0.003 (−0.01, 0.02)
IV	0.02 (−0.04, 0.09)	−0.004 (−0.02, 0.01)	0.02 (−0.05, 0.08)	0.0004 (−0.01, 0.01)
L5 time	−0.04 (−0.10, 0.01)	−0.004 (−0.02, 0.01)	−0.03 (−0.09, 0.03)	−0.01 (−0.02, 0.01)
M10 time	0.004 (−0.06, 0.06)	−0.01 (−0.03, 0.002)	0.01 (−0.06, 0.07)	−0.01 (−0.02, 0.004)

– = significant interaction, main effect not reported (see tables below). Model 1 adjusted for baseline age, sex, race, years of education, time, baseline age*time, sex*time, race*time, and years of education*time. Model 2 adjusted for Model 1 covariates, in addition to baseline BMI, sleep medication use, APOE e4 status, depression symptoms (minus the sleep item), cardiovascular disease risk, smoking status, BMI*time, cardiovascular disease risk*time, smoking status*time, sleep medication*time, APOE e4 status*time, and depression symptoms (minus the sleep item)*time. ^†^*p* < 0.10.

### Visuospatial performance

There were no significant main effects of CRARs on visuospatial performance (Table 9). Age moderated associations of mesor with respect to visuospatial performance cross sectionally (*p*-value for interaction = 0.002). Model-derived point estimates indicate that at age 85, higher mesor was associated with poorer visuospatial performance; these associations were not evident at ages 65 or 75 (Table 11).

**TABLE 9:** Associations of circadian metrics with visuospatial ability, estimated beta (95% CI).

	Model 1		Model 2	
	Baseline cross-sectional effect	Effect on cognitive change	Baseline cross-sectional effect	Effect on cognitive change
**Cosinor metrics**				
Amplitude	–	–	–	–
Mesor	–	–	–	–
Acrophase	−0.02 (−0.09, 0.04)	0.005 (−0.01, 0.02)	−0.03 (−0.09, 0.04)	0.004 (−0.01, 0.02)
**Non-Parametric**				
RA	0.04 (−0.02, 0.11)	−0.01^†^ (−0.03, 0.002)	0.04 (−0.02, 0.11)	−0.01 (−0.03, 0.004)
IS	–	–	–	–
IV	–	–	–	–
L5 time	−0.01 (−0.07, 0.04)	0.005 (−0.01, 0.02)	−0.01 (−0.07, 0.06)	0.002 (−0.01, 0.02)
M10 time	−0.002 (−0.06, 0.06)	0.002 (−0.01, 0.02)	−0.01 (−0.07, 0.06)	0.002 (−0.01, 0.02)

– = significant interaction, main effect not reported (see tables below). Model 1 adjusted for baseline age, sex, race/ethnicity, years of education, time, baseline age*time, sex*time, race*time, and years of education*time. Model 2 adjusted for Model 1 covariates, in addition to baseline BMI, sleep medication use, APOE e4 status, depression symptoms (minus the sleep item), cardiovascular disease risk, smoking status, BMI*time, cardiovascular disease risk*time, smoking status*time, sleep medication*time, APOE e4 status*time, and depression symptoms (minus the sleep item)*time. ^†^*p* < 0.10.

We found several significant sex by CRAR interactions with visuospatial performance (Table 12). There was an interaction between sex and IS in predicting visuospatial performance cross-sectionally (*p*-value for interaction = 0.046) such that greater IS was associated with better visuospatial performance among men, but not women.

We observed a significant longitudinal interaction of race with IV and visuospatial performance (*p*-value for interaction = 0.014) (Table 13). Greater IV was associated with greater declines in visuospatial performance in Black adults, but not White adults.

Further, APOE e4 carrier status moderated the cross-sectional association of amplitude with visuospatial performance (*p*-value for interaction = 0.004; Table 14) such that greater amplitude was associated with better visuospatial performance among e4 carriers but not non-carriers.

### Attention

Although there were no significant main effects of CRARs on attention (Table 10), we observed significant interactions of sex with IV (*p*-value for interaction = 0.032) and amplitude (*p*-value for interaction = 0.033), although sex-specific point estimates were not significant (Table 12).

**TABLE 10:** Associations of circadian metrics with attention, estimated beta (95% CI).

	Model 1		Model 2	
	Baseline cross-sectional effect	Effect on cognitive change	Baseline cross-sectional effect	Effect on cognitive change
**Cosinor metrics**				
Amplitude	–	–	–	–
Mesor	–	–	–	–
Acrophase	−0.02 (−0.09, 0.04)	0.003 (−0.01, 0.02)	−0.02 (−0.09, 0.04)	0.004 (−0.01, 0.02)
**Non-parametric**				
RA	0.02 (−0.04, 0.08)	0.002 (−0.01, 0.02)	0.01 (−0.06, 0.07)	0.001 (−0.01, 0.02)
IS	0.05^†^ (−0.01, 0.11)	−0.003 (−0.02, 0.01)	0.05 (−0.02, 0.11)	−0.01 (−0.02, 0.01)
IV	–	–	–	–
L5 time	−0.004 (−0.06, 0.05)	†−0.01 (−0.02, 0.001)	0.003 (−0.05, 0.06)	−0.01^†^ (−0.02, 0.002)
M10 time	−0.01 (−0.07, 0.05)	0.01 (−0.01, 0.02)	−0.01 (−0.07, 0.05)	0.01 (−0.01, 0.02)

– = significant interaction, main effect not reported (see tables below). Model 1 adjusted for baseline age, sex, race, years of education, time, baseline age*time, sex*time, race*time, and years of education*time. Model 2 adjusted for Model 1 covariates, in addition to baseline BMI, sleep medication use, APOE e4 status, depression symptoms (minus the sleep item), cardiovascular disease risk, smoking status, BMI*time, cardiovascular disease risk*time, smoking status*time, sleep medication*time, APOE e4 status*time, and depression symptoms (minus the sleep item)*time. ^†^*p* < 0.10.

**TABLE 11:** Age: significant cross-sectional and longitudinal interactions with CRARs on cognition, estimated beta (95% CI).

Cross-sectional associations	65 years	75 years	85 years	Interaction *p*-value
**Visuospatial performance**				
Mesor	0.05 (−0.03, 0.13)	−0.05 (−0.12, 0.02)	−0.15** (−0.25, −0.05)	0.002

**Longitudinal associations**	65 years*time	75 years*time	85 years*time	**Interaction *p*-value**

**Language**				
Amplitude	−0.01 (−0.03, 0.004)	0.004 (−0.01, 0.02)	0.02* (0.003, 0.04)	0.006
Mesor	−0.03** (−0.04, −0.01)	0.0005 (−0.01, 0.01)	0.03** (0.01, 0.05)	0.0001
IS	−0.01 (−0.02, 0.01)	0.01 (−0.01, 0.02)	0.02* (0.002, 0.04)	0.036
IV	0.02* (0.004, 0.04)	−0.001 (−0.01, 0.01)	−0.02* (−0.04, −0.004)	0.0005

*p < 0.05; **p < 0.01. Baseline age was modeled as continuous variable. Estimates of associations at specific ages of 65, 75, and 85 were derived from models. Models adjusted for baseline age, sex, race, years of education, BMI, cardiovascular disease risk, sleep medication, APOE e4 status, depression symptoms (minus the sleep item), smoking status, time, baseline age*time, sex*time, race*time, years of education*time, BMI*time, cardiovascular disease risk*time, sleep medication*time, APOE e4 status*time, depression symptoms (minus the sleep item)*time, and smoking status*time.

**TABLE 12:** Sex: significant cross-sectional interactions with CRARs on cognition, estimated beta (95% CI).

Cross-sectional associations	Men (n = 199)	Women (n = 223)	Interaction *p*-value
**Visuospatial performance**			
IS	0.10* (0.01, 0.18)	−0.04 (−0.14, 0.06)	0.046
**Attention**			
Amplitude	0.07 (−0.01, 0.16)	−0.07 (−0.16, 0.03)	0.033
IV	−0.06 (−0.14, 0.02)	0.07 (−0.03, 0.17)	0.032

*p < 0.05. Models adjusted for baseline age, sex, race, years of education, BMI, cardiovascular disease risk, sleep medication, APOE e4 status, depression symptoms (minus the sleep item), smoking status, time, baseline age*time, sex*time, race*time, years of education*time, BMI*time, cardiovascular disease risk*time, sleep medication*time, APOE e4 status*time, depression symptoms (minus the sleep item)*time, and smoking status*time.

**TABLE 13:** Race: significant longitudinal interactions with CRARs on cognition, estimated beta (95% CI).

Longitudinal Associations	White adults (n = 302)	Black adults (n = 93)	Interaction *p*-value
**Verbal memory**			
M10 time	0.06 (−0.03, 0.15)	−0.12 (−0.26, 0.03)	0.040
**Language**			
Mesor	0.01 (−0.01, 0.02)	−0.04** (−0.07, −0.01)	0.004
IV	−0.01 (−0.02, 0.01)	0.04* (0.004, 0.07)	0.019
M10 time	−0.01 (−0.02, 0.01)	0.03* (0.01, 0.06)	0.005
**Visuospatial performance**			
IV	0.01 (−0.01, 0.02)	−0.05** (−0.09, −0.01)	0.014

*p < 0.05; **p < 0.01. Models adjusted for baseline age, sex, race, years of education, BMI, cardiovascular disease risk, sleep medication, APOE e4 status, depression symptoms (minus the sleep item), smoking status, time, baseline age*time, sex*time, race*time, years of education*time, BMI*time, cardiovascular disease risk*time, sleep medication*time, APOE e4 status*time, depression symptoms (minus the sleep item)*time, and smoking status*time.

**TABLE 14:** APOE e4 carrier status: significant cross-sectional and longitudinal interactions with CRARs on cognition, beta estimate (95% CI).

Cross-sectional associations	APOE e4−(n = 291)	APOE e4+(n = 103)	Interaction *p*-value
**Executive function**			
IS	−0.02 (−0.09, 0.06)	0.17* (0.03, 0.30)	0.015
**Attention**			
Mesor	−0.04 (−0.11, 0.04)	0.01 (−0.005, 0.23)	0.033
**Language**			
Mesor	−0.002 (−0.0**7**, 0.07)	0.18** (0.07, 0.30)	0.008
**Visuospatial performance**			
Amplitude	−0.03 (−0.11, 0.05)	0.22** (0.07, 0.37)	0.004

**Longitudinal associations**	APOE e4−*time (n = 291)	APOE e4 + *time (n = 103)	**Interaction *p*-value**

**Language**			
Mesor	0.01 (−0.01, 0.02)	−0.03* (−0.05, −0.002)	0.024

*p < 0.05; **p < 0.01. Models adjusted for baseline age, sex, race, years of education, BMI, cardiovascular disease risk, sleep medication, APOE e4 status, depression symptoms (minus the sleep item), smoking status, time, baseline age*time, sex*time, race*time, years of education*time, BMI*time, cardiovascular disease risk*time, sleep medication*time, APOE e4 status*time, depression symptoms (minus the sleep item)*time, and smoking status*time.

APOE e4 carrier status moderated the cross-sectional association of mesor with attention (*p*-value for interaction = 0.033), though these point estimates were also not significant (Table 14).

### Sensitivity analyses

When we analyzed the relations between CRARs binned at 60-min and cognitive performance and change, there were fewer significant main effects and interactions (Supplementary Tables 1–9).

## Discussion

Although previous work has examined associations of CRARs with cognitive functioning (Oosterman et al., 2009; Luik et al., 2015), no studies to our knowledge have explored interactions of parametric and non-parametric CRAR indices with age, sex, race, or APOE e4 status, with respect to performance and change in multiple cognitive domains. Several main effect associations of CRARs with cognitive outcomes were observed. For example, lower rhythm strength (i.e., interdaily stability) was associated with greater declines in memory, which parallels findings from Xiao et al. (2022a) in which individuals with lower interdaily stability were more likely to develop incident cognitive impairments. In minimally adjusted models, we also observed a marginally significant association of greater rhythm strength (i.e., amplitude) with slower declines in executive function, paralleling previous findings observed in work conducted by Walsh et al. (2014). There were few main effects, however, in comparison to the numerous interactions we observed between participant characteristics (i.e., age, sex, race, and APOE e4 carrier status) and CRARs with respect to cognitive outcomes. These findings highlight the complexity of CRAR-cognition associations and the importance of considering these participant characteristics when studying links of CRARs with cognitive function and decline.

We observed several interactions of age and CRARs, with consistently opposite associations observed at age 65 vs. age 85. For example, higher mesor was associated with poorer visuospatial performance at ages 85, but not ages 65 or 75. Measures of greater rhythm strength (i.e., amplitude, mesor), greater consistency (i.e., IS), and lower fragmentation (i.e., IV) were associated with slower language declines at age 85; however, some of these associations were null or reversed at age 65. Although these findings are consistent with prior research linking lower CRAR strength to incident MCI and dementia in women with a mean age of 83 (Tranah et al., 2011), it is unclear why the effect would be opposite at an earlier age. As one possible explanation, in the younger old, among whom neurodegeneration has more recently begun, CRARs may have effects on cognitive performance that are independent of neurodegenerative disease. In the older old, however, among whom neurological deficits may be more advanced, neurological changes may be a shared cause of both CRAR alterations and cognitive impairment. This may account for the differences we observed. Alternatively, participants who have been retained in the study over time may be healthy compared to those who dropped out (potentially for health-related reasons). In other words, survival bias may account for some of the differences in CRAR-cognition associations among age groups. On the other hand, if CRARs are in fact causing cognitive decline, our findings would suggest that intervening on CRARs may have different effects on cognition at different ages. Further work in this area is needed, including studies investigating mechanisms that link CRARs to cognition across the life course.

There was also evidence that associations of CRARs and cognitive performance differed as a function of sex. For example, we found that greater rhythm strength (as measured by interdaily stability) was associated with better visuospatial performance in men, but not women. Sex differences in cognitive performance have been identified previously (Levine et al., 2021). For example, in a prior BLSA study, compared to cognitively healthy older women, their male counterparts performed better on visuospatial tasks and more poorly in other cognitive domains (e.g., memory, executive function), and showed greater declines in visuospatial ability (McCarrey et al., 2016). We are unaware, however, of prior studies that have examined sex-CRAR interactions with regard to cognitive domain trajectories to which we can compare our findings. However, there is evidence that poorer sleep is more strongly associated with poorer cognitive performance among women than among men (Rangtell et al., 2019). Given the aforementioned sex differences in dementia and our findings of sex differences in CRAR-cognition links, further research is needed investigating sex differences in the associations of CRARs with cognitive outcomes.

We also found that race moderated associations of CRARs with change over time in language and visuospatial performance, such that these associations were stronger among Black relative to White participants. For example, lower rhythm fragmentation (i.e., lower IV) predicted slower declines in visuospatial performance in Black participants, but not White participants. Several studies demonstrate a higher incidence of dementia among Black older adults relative to White older adults (Tang et al., 2001; Mayeda et al., 2016), and differences in the incidence rates of dementia may be partially driven by documented racial differences in CRARs. For example, compared to White older adults, Black older adults tend to have weaker rhythm strength (i.e., lower amplitude, lower IS) (Mitchell et al., 2017). Differences in CRARs among Black adults and White adults may be due to a host of socioeconomic factors (e.g., occupation, neighborhood conditions) emanating from historical and contemporary forms of structural racism and discrimination that likely increase psychological stress, lead to physiological arousal, and subsequently adversely affect sleep/wake patterns (Jackson et al., 2013; Slopen et al., 2016; Gaston et al., 2020; Johnson et al., 2021). Importantly, our findings suggest that circadian rhythmicity is a more important predictor of change over time in cognitive functioning among Black, compared to White, middle-aged and older adults. Interventions aimed at improving cognitive health in Black adults should also consider targeting CRARs, as well as modifiable social and environmental exposures (e.g., poverty, discrimination), in this population. Such an approach has the potential to reduce widespread health disparities in aging-related diseases (Jackson et al., 2021).

We observed numerous interactions of APOE e4 carrier status with CRARs in relation to cognitive performance and decline such that the association of CRARs with cognitive outcomes were stronger among APOE e4 carriers relative to non-carriers. The APOE e4 allele has been linked to poorer cognitive performance and greater cognitive decline in older adults (Nilsson et al., 2006; Brewster et al., 2014; Zhen et al., 2017; Todd et al., 2018; Gharbi-Meliani et al., 2021), and APOE e4 may increase vulnerability to cognitive effects of Alzheimer’s disease (AD) pathology (Weigand et al., 2021). Other research conducted in the BLSA found differences in the association of the APOE e4 and cognition based on age and sex (Williams et al., 2019). To our knowledge, no studies have examined APOE genotype-CRAR interactions with change in cognition over time. However, findings from animal models indicate that APOE e4 allelic variation in mice may contribute to alterations in circadian rhythms (Graybeal et al., 2015). Regardless, our findings suggest that associations of CRARs (i.e., weaker rhythm strength) and cognitive decline are more robust among APOE e4 carriers. Although the mechanisms of this interaction are unclear, findings may reflect a dual vulnerability in which the combination of higher genetic liability for AD and specific CRAR patterns may confer greater risk for cognitive decline. Further studies are needed to investigate the extent to which persons at high genetic risk for AD are susceptible to negative cognitive effects of circadian disruption. Notably, prior research in the BLSA has linked APOE genotype to self-reported measures of shorter sleep duration (Spira et al., 2017). Further studies investigating associations of APOE and other AD risk genes with actigraphic sleep and CRARs are needed.

The present study has important strengths. To our knowledge, it is the first study to investigate cross-sectional and longitudinal associations of parametric and non-parametric actigraphic CRAR metrics with performance and change across multiple cognitive domains in middle-aged and older adults, and the first to explore whether these associations differ as a function of age, sex, race, and APOE e4 carrier status. Moderation analyses revealed that associations of CRARs with cognitive performance and decline were generally stronger among older adults aged 85, women, Black participants, and APOE e4 carriers. Future work is needed to examine the effects of interventions targeting circadian rhythms [e.g., bright light exposure (Klerman et al., 2001), melatonin (Zisapel, 2018), scheduled activity (Safi and Hodgson, 2014)] on cognitive performance and change in older adults in general, and in these subgroups in particular, given our findings suggest they may be more vulnerable to cognitive effects of circadian alterations. These effects may include dementia due to various etiologies, including AD. Future studies with larger samples are needed to replicate our observed pattern of results, but with MCI and dementia as outcomes and inclusion of AD biomarkers to elucidate the potential mediating neurodegenerative disease process.

Despite these strengths, this study has limitations that must be considered. Because this is the first cross-sectional and prospective study of both parametric and non-parametric CRAR metrics and multiple domains of cognition and key interactions, we erred on the side of multiple testing to avoid Type II error. Consequently, our results may include false positives due to multiple comparisons. Second, our sample consisted primarily of participants with a high level of education, all of whom were very healthy at enrollment; thus, results may not generalize to the broader population of older adults. Both limitations necessitate replication of our findings in independent cohorts more representative of the general population of middle-aged and older adults. In addition, the number of days of actigraphy data collection was relatively small. Future research should collect actigraphic data over a longer duration of time, which would allow for more accurate estimates of participants’ CRAR parameters. In addition, future work should examine interactions of actigraphic sleep parameters and CRARs in predicting cognition over time.

In sum, our findings demonstrate that CRARs are generally more strongly associated with cognitive performance and change among older individuals, women, Black adults, and APOE e4 carriers. Future research is needed to determine the mechanisms underlying these differential associations, and whether individuals in these populations are more likely than those from other populations to derive cognitive benefits from CRAR-based interventions.

## Data availability statement

The data underlying this study are available on request from the BLSA website (https://www.blsa.nih.gov). All requests undergo a review process performed by the BLSA Data Sharing Proposal Review Committee. Requests to access the datasets should be directed to https://www.blsa.nih.gov.

## Ethics statement

The studies involving human participants were reviewed and approved by National Institutes of Health Institutional Review Board. The patients/participants provided their written informed consent to participate in this study.

## Author contributions

JR conducted the literature review and took a lead role in writing the manuscript. YA provided guidance regarding the statistical analyses. LH and AA provided their expertise on circadian rest activity rhythms and helped in the framing of the introduction. VZ oversaw the processing and cleaning of the actigraphic data. SW was involved in exporting the actigraphic data and pre-processing of the actigraphic data. LF, ES, SR, MW, and JS provided feedback on the study design and conceptualization. CJ provided her expertise on health disparities in sleep and other health outcomes. AS conceived of the study and edited the manuscript. All authors provided intellectual input and feedback on the manuscript and approved the submitted version.

## Footnotes

1
https://cran.r-project.org/web/packages/cosinor/cosinor.pdf

2
https://cran.r-project.org/web/packages/ActCR/index.html
