# Supplementary material for: Associations of circadian rest/activity rhythms with cognition in middle-aged and older adults: Demographic and genetic interactions
Source: Front Neurosci. 2022 Oct 12;16:952204. doi: 10.3389/fnins.2022.952204 (PMC9597505; doi:10.3389/fnins.2022.952204)
Supplement: Supplementary file 1 [file Data_Sheet_1.PDF]

Supplementary Table 1. Associations of Circadian Metrics with Memory, estimated beta (95% CI)

	Model 1		Model 2	
	**Baseline Cross-Sectional Effect**	**Effect on Cognitive Change**	**Baseline Cross-Sectional Effect**	**Effect on Cognitive Change**
Cosinor Metrics				
**Amplitude**	0.02 (-0.06, 0.10)	†0.02 (-0.002, 0.04)	0.02 (-0.07, 0.10)	0.02 (-0.004, 0.03)
**Mesor**	-0.002 (-0.08, 0.07)	0.0001 (-0.02, 0.02)	-0.001 (-0.08, 0.08)	-0.002 (-0.02, 0.02)
**Acrophase**	0.03 (-0.05, 0.10)	-0.01 (-0.03, 0.01)	0.02 (-0.06, 0.10)	-0.01 (-0.03, 0.01)
Non-Parametric				
**RA**	0.04 (-0.04, 0.12)	0.01 (-0.01, 0.03)	0.05 (-0.03, 0.13)	0.01 (-0.01, 0.03)
**IS**	0.02 (-0.05, 0.10)	**0.03** (0.01, 0.05)**	0.02 (-0.06, 0.10)	**0.03** (0.01, 0.05)**
**IV**	-0.02 (-0.09, 0.06)	**-0.02* (-0.04, -0.002)**	-0.02 (-0.09, 0.06)	-0.02† (-0.04, 0.001)
**L5 time**	**-0.07* (-0.14, -0.002)**	-0.01 (-0.02, 0.01)	**-0.08* (-0.15, -0.01)**	-0.003 (-0.02, 0.01)
**M10 time**	0.02 (-0.05, 0.09)	-0.005 (-0.02, 0.01)	0.01 (-0.06, 0.08)	-0.003 (-0.02, 0.01)

Notes. †p < 0.10; *p <.05; **p <.01; ***p <.001; --= significant interaction (see tables below).
Model 1 adjusted for baseline age, sex, race, years of education, time, baseline age*time, sex*time, race*time, and years of education*time.
Model 2 adjusted for Model 1 covariates, in addition to BMI, cardiovascular disease risk, smoking status, sleep medication use, APOE e4 status, depression symptoms (minus the sleep item), BMI*time, cardiovascular disease risk*time, smoking status*time, sleep medication use*time, APOE e4 status*time, and depression symptoms (minus the sleep item)*time.

Supplementary Table 2. Associations of Circadian Metrics with Executive Function, estimated beta (95% CI)

	**Model 1**		**Model 2**	
	**Baseline Cross-Sectional Effect**	**Effect on Cognitive Change**	**Baseline Cross-Sectional Effect**	**Effect on Cognitive Change**
Cosinor Metrics				
**Amplitude**	-0.02 (-0.09, 0.04)	†0.01 (-0.001, 0.03)	-0.02 (-0.09, 0.05)	0.01 (-0.01, 0.03)
**Mesor**	-0.05 (-0.11, 0.01)	0.003 (-0.01, 0.02)	-0.05 (-0.12, 0.01)	0.002 (-0.01, 0.02)
**Acrophase**	0.001 (-0.06, 0.07)	-0.01 (-0.03, 0.003)	0.01 (-0.06, 0.07)	-0.01 (-0.03, 0.01)
Non-Parametric				
**RA**	0.03 (-0.04, 0.10)	0.006 (-0.01, 0.02)	0.04 (-0.03, 0.10)	0.003 (-0.01, 0.02)
**IS**	--	--	--	--
**IV**	0.003 (-0.06, 0.06)	-0.01 (-0.03, 0.004)	-0.01 (-0.07, 0.05)	-0.01 (-0.02, 0.01)
**L5 time**	†-0.05 (-0.11, 0.005)	-0.004 (-0.01, 0.01)	-0.04 (-0.10, 0.02)	-0.01 (-0.02, 0.01)
**M10 time**	--	--	--	--

Notes. †p < 0.10; *p <.05; **p <.01; -- = significant interaction (see tables below).
Model 1 adjusted for baseline age, sex, race, years of education, time, baseline age*time, sex*time, race*time, and years of education*time.
Model 2 adjusted for Model 1 covariates, in addition to BMI, cardiovascular disease risk, smoking status, sleep medication use, APOE e4 status, depression symptoms (minus the sleep item), BMI*time, cardiovascular disease risk*time, smoking status*time, sleep medication use*time, APOE e4 status*time, and depression symptoms (minus the sleep item)*time.

Supplementary Table 3. Associations of Circadian Metrics with Language, estimated beta (95% CI)

	**Model 1**		**Model 2**	
	**Baseline Cross-Sectional Effect**	**Effect on Cognitive Change**	**Baseline Cross-Sectional Effect**	**Effect on Cognitive Change**
Cosinor Metrics				
**Amplitude**	--	--	--	--
**Mesor**	--	--	--	--
**Acrophase**	0.01 (-0.05, 0.07)	0.003 (-0.01, 0.01)	0.01 (-0.05, 0.08)	0.002 (-0.01, 0.01)
Non-Parametric				
**RA**	--	--	--	--
**IS**	--	--	--	--
**IV**	--	--	--	--
**L5 time**	†-0.05 (-0.10, 0.01)	0.002 (-0.01, 0.01)	-0.04 (-0.09, 0.02)	0.002 (-0.01, 0.01)
**M10 time**	--	--	--	--

Notes. †p < 0.10; *p <.05; **p <.01; -- = significant interaction (see tables below).
Model 1 adjusted for baseline age, sex, race, years of education, time, baseline age*time, sex*time, race*time, and years of education*time.
Model 2 adjusted for Model 1 covariates, in addition to BMI, cardiovascular disease risk, smoking status, sleep medication use, APOE e4 status, depression symptoms (minus the sleep item), BMI*time, cardiovascular disease risk*time, smoking status*time, sleep medication use*time, APOE e4 status*time, and depression symptoms (minus the sleep item)*time.

Supplementary Table 4. Associations of Circadian Metrics with Visuospatial Skills, estimated beta (95% CI)

	**Model 1**		**Model 2**	
	**Baseline Cross-Sectional Effect**	**Effect on Cognitive Change**	**Baseline Cross-Sectional Effect**	**Effect on Cognitive Change**
Cosinor Metrics				
**Amplitude**	--	--	--	--
**Mesor**	--	--	--	--
**Acrophase**	-0.03 (-0.09, 0.04)	0.004 (-0.01, 0.02)	-0.03 (-0.09, 0.04)	0.004 (-0.01, 0.02)
Non-parametric				
**RA**	0.04 (-0.03, 0.11)	-0.01† (-0.03, 0.001)	0.05 (-0.02, 0.11)	-0.01 (-0.03, 0.004)
**IS**	0.04 (-0.03, 0.10)	0.01 (-0.005, 0.03)	0.05 (-0.02, 0.12)	0.01 (-0.003, 0.03)
**IV**	-0.04 (-0.10, 0.03)	0.001 (-0.02, 0.02)	-0.05 (-0.11, 0.02)	-0.0002 (-0.02, 0.02)
**L5 time**	-0.01 (-0.07, 0.04)	0.01 (-0.01, 0.02)	-0.01 (-0.07, 0.05)	0.002 (-0.01, 0.01)
**M10 time**	--	--	--	--

Notes. †p < 0.10; *p <.05; **p <.01; -- = significant interaction (see tables below).

Model 1 adjusted for baseline age, sex, race, years of education, time, baseline age*time, sex*time, race*time, and years of education*time.

Model 2 adjusted for Model 1 covariates, in addition to BMI, cardiovascular disease risk, smoking status, sleep medication use, APOE e4 status, depression symptoms (minus the sleep item), BMI*time, cardiovascular disease risk*time, smoking status*time, sleep medication use*time, APOE e4 status*time, and depression symptoms (minus the sleep item)*time.

Supplementary Table 5. Associations of Circadian Metrics with Attention, estimated beta (95% CI)

	**Model 1**		**Model 2**	
	**Baseline Cross-Sectional Effect**	**Effect on Cognitive Change**	**Baseline Cross-Sectional Effect**	**Effect on Cognitive Change**
Cosinor Metrics				
**Amplitude**	--	--	--	--
**Mesor**	--	--	--	--
**Acrophase**	-0.02 (-0.09, 0.04)	0.003 (-0.01, 0.02)	-0.02 (-0.09, 0.04)	0.004 (-0.01, 0.02)
Non-Parametric				
**RA**	0.02 (-0.04, 0.08)	0.002 (-0.01, 0.02)	0.01 (-0.06, 0.07)	0.001 (-0.01, 0.02)
**IS**	†0.06 (-0.004, 0.12)	-0.002 (-0.02, 0.01)	†0.06 (-0.01, 0.12)	-0.01 (-0.02, 0.01)
**IV**	-0.02 (-0.08, 0.04)	0.01 (-0.01, 0.02)	-0.01 (-0.08, 0.05)	0.01 (-0.01, 0.02)
**L5 time**	-0.01 (-0.07, 0.04)	†-0.01 (-0.02, 0.001)	0.001 (-0.05, 0.06)	†-0.01 (-0.02, 0.001)
**M10 time**	-0.02 (-0.08, 0.03)	0.01 (-0.01, 0.02)	-0.02 (-0.08, 0.04)	0.01 (-0.01, 0.02)

Notes. †p < 0.10; *p <.05; **p <.01; -- = significant interaction (see tables below).
Model 1 adjusted for baseline age, sex, race, years of education, time, baseline age*time, sex*time, race*time, and years of education*time.
Model 2 adjusted for Model 1 covariates, in addition to BMI, cardiovascular disease risk, smoking status, sleep medication use, APOE e4 status, depression symptoms (minus the sleep item), BMI*time, cardiovascular disease risk*time, smoking status*time, sleep medication use*time, APOE e4 status*time, and depression symptoms (minus the sleep item)*time.

Supplementary Table 6. Age: Significant Cross-Sectional and Longitudinal Interactions with Circadian Rhythm on Cognition, estimated beta (95% CI)

Cross-sectional Associations	**65 years**	**75 years**	**85 years**	**Interaction p-value**
Visuospatial ability				
**Mesor**	0.05 (-0.03, 0.13)	-0.05 (-0.12, 0.02)	**-0.15** (-0.25, -0.05)**	0.002
Longitudinal Associations	**65 years*time**	**75 years*time**	**85 years*time**	**Interaction p-value**
Executive Function				
**M10 time**	**-0.02* (-0.04, -0.004)**	-0.01 (-0.02, 0.01)	0.01 (-0.01, 0.03)	0.044
Language				
**Amplitude**	-0.01 (-0.03, 0.004)	0.004 (-0.01, 0.02)	**0.02* (0.003, 0.04)**	0.006
**Mesor**	**-0.03** (-0.04, -0.01)**	0.0005 (-0.01, 0.01)	**0.03** (0.01, 0.05)**	0.0001
**IS**	-0.005 (-0.02, 0.01)	0.01 (-0.01, 0.02)	0.02 (-0.0001, 0.04)	0.050
**IV**	0.01 (-0.005, 0.03)	-0.01 (-0.02, 0.01)	**-0.02** (-0.04, -0.01)**	0.003

Notes. *p <.05; **p <.01.

Models adjusted for baseline age, sex, race, years of education, BMI, cardiovascular disease risk, sleep medication, APOE e4 status, depression symptoms (minus the sleep item), smoking status, time, baseline age*time, sex*time, race*time, years of education*time, BMI*time, cardiovascular disease risk*time, sleep medication*time, APOE e4 status*time, smoking status*time, and depression symptoms (minus the sleep item)*time.

Supplementary Table 7. Sex: Significant Cross-Sectional and Longitudinal Interactions with Circadian Rhythm on Cognition, estimated beta (95% CI)

Cross-sectional Associations	**Men**	**Women**	**Interaction p-value**
Attention			
**Amplitude**	0.07 (-0.01, 0.16)	-0.07 (-0.17, 0.03)	**0.032**
Longitudinal Associations	**Men*time N = 201**	**Women*time N = 223**	**Interaction p-value**
Language			
**RA**	0.01 (-0.002, 0.03)	-0.02 (-0.04, 0.01)	0.043
**M10 time**	-0.01 (-0.03, 0.01)	**0.02* (0.0001, 0.03)**	0.019
Visuospatial ability			
**M10 time**	-0.01 (-0.03, 0.01)	0.02 (-0.001, 0.04)	0.039

Notes. *p <.05; **p <.01.

Models adjusted for baseline age, sex, race, years of education, BMI, cardiovascular disease risk, sleep medication, APOE e4 status, depression symptoms (minus the sleep item), smoking status, time, baseline age*time, sex*time, race*time, years of education*time, BMI*time, cardiovascular disease risk*time, sleep medication*time, APOE e4 status*time, smoking status*time, and depression symptoms (minus the sleep item)*time.

Supplementary Table 8. Race: Significant Interactions with Circadian Rhythm on Cognition, estimated beta (95% CI)

Longitudinal Associations	**White adults*time N = 302**	**Black adults*time N = 93**	**Interaction p-value**
Language			
**Mesor**	0.01 (-0.01, 0.02)	**-0.04** (-0.07, -0.01)**	0.004
**IV**	-0.02 (-0.03, 0.004)	0.03 (-0.01, 0.07)	0.039
**M10 time**	-0.01 (-0.02, 0.01)	**0.03** (0.01, 0.06)**	0.004

Notes. *p <.05; **p <.01.

Models adjusted for baseline age, sex, race, years of education, BMI, cardiovascular disease risk, sleep medication, APOE e4 status, depression symptoms (minus the sleep item), smoking status, time, baseline age*time, sex*time, race*time, years of education*time, BMI*time, cardiovascular disease risk*time, sleep medication*time, APOE e4 status*time, smoking status*time, and depression symptoms (minus the sleep item)*time.

Supplementary Table 9. APOE e4 Carrier Status: Significant Cross-sectional and Longitudinal Interactions with Circadian Rhythm on Cognition, beta estimate (95% CI)

Cross-sectional Associations	**APOE e4 - N = 291**	**APOE e4 + N = 103**	**Interaction p-value**
Executive Function			
**IS**	-0.0004 (-0.08, 0.07)	**0.17** (0.04, 0.29)**	**0.021**
Attention			
**Mesor**	-004 (-0.11, 0.04)	0.11 (-0.01, 0.23)	**0.034**
Language			
**Mesor**	-0.002 (-0.07, 0.07)	**0.19** (0.07, 0.30)**	**0.008**
Visuospatial ability			
**Amplitude**	-0.03 (-0.11, 0.05)	**0.22* (0.07, 0.37)**	**0.033**
Longitudinal Associations	**APOE e4 -*time N = 291**	**APOE e4 +*time N = 103**	**Interaction p-value**
Language			
**Mesor**	0.01 (-0.01, 0.02)	**-0.03* (-0.05, -0.002)**	**0.024**

Notes. *p <.05; **p <.01.

Models adjusted for baseline age, sex, race, years of education, BMI, cardiovascular disease risk, sleep medication, APOE e4 status, depression symptoms (minus the sleep item), smoking status, time, baseline age*time, sex*time, race*time, years of education*time, BMI*time, cardiovascular disease risk*time, sleep medication*time, APOE e4 status*time, smoking status*time, and depression symptoms (minus the sleep item)*time.
